# Complex Social Structure of an Endangered Population of Bottlenose Dolphins (*Tursiops truncatus*) in the Aeolian Archipelago (Italy)

**DOI:** 10.1371/journal.pone.0114849

**Published:** 2014-12-10

**Authors:** Monica F. Blasi, Luigi Boitani

**Affiliations:** 1 Filicudi WildLife Conservation, Località Stimpagnato Filicudi, 98055, Lipari (ME), Italy; 2 Department of Biology and Biotechnologies, University ‘La Sapienza’, Viale dell' Università 32, 00185, Rome, Italy; University of Missouri, United States of America

## Abstract

We investigated social structure and association patterns for a small population of Mediterranean bottlenose dolphins, *Tursiops truncatus*, inhabiting the Aeolian Archipelago (southern Italy). Specifically we evaluate the role of sex and age composition, residency patterns and interaction with trammel nets on this social organization. Association data for 23 regularly sighted individuals were obtained from summer photoidentification surveys collected from 2005–2012. Using a combined cluster and social network analysis approach, we found associations between dolphins were hierarchically structured, where two mixed-sex social units were subdivided into smaller temporarily dynamic groups. We found non-random and long-term preferred associations in the population; however, the degree of social cohesion, residence pattern and interaction with trammel nets differed considerably between the two social units. Six of eight females occurred in the more resident social unit-1; in addition, social unit-1 individuals had significantly stronger associations, higher preferred associates, lived in larger groups and occurred less frequently with trammel nets. Nine of eleven males were clustered in social unit-2 and five of these males, interacting with trammel nets, formed small groups and preferred associations. We propose that female and male groups associate in the study area during the breeding season and that some males choose to interact with reproductive females forming a distinct but interrelated social unit. Other males may be associating in a larger fission-fusion network, which consists of dolphins that appear to temporarily join the network from the coastal population. We cannot exclude that some males specialized in trammel net foraging, suggesting that this foraging technique may favor a solitary lifestyle. Large group sizes and high degree of social cohesion for females could be an indication of greater protection and more efficiency in detecting, deterring or repelling anthropogenic pressures. Most likely dolphins' social organization depends on a combination of socio-ecological, demographic and anthropogenic factors.

## Introduction

Bottlenose dolphins (*Tursiops truncatus*), like many mammal species [1], [2], [3], live in fission–fusion societies where individuals join and leave groups on a flexible basis, with group composition and size changing frequently on small spatial and temporal scales [4]. High rates of group composition changes in *fission–fusion* societies are thought to be an adaptive response to the dynamic interaction of ecological variables [5].

Several factors potentially affect the social structure of bottlenose dolphins, including predation risk [6], habitat structure [7], [8], [9], prey distribution [6], [10], cultural transmission [11], [12], [13], breeding success [14], male competition [15], [16], [17], [18], and risk of infanticide [19], [20], [21]. The stability of social relationships in a population, however, is expected to increase when the benefits of stable, cooperative relationships balance the costs, such as increased resource competition and socially transmitted parasites [22]. Sexual segregation is believed to be the basic social framework of some bottlenose dolphin populations (e.g. *T. aduncus* in eastern Australia) where grouping patterns reflect sex-specific behavioural strategies [23]. In some wild populations, sexually mature male bottlenose dolphins form first-order alliances in pairs, trios, and second-order super-alliances, which cooperate by pursuing distinct alliance strategies to monopolize females in reproductive condition [15], [16], [17], [18]. However, other studies have shown that male alliances may not occur in all bottlenose dolphin populations and the degree of sexual segregation and the proportion of mixed-sex groups can be geographically very variable [24], [25], [26], [27]. Females may be highly social or even solitary (e.g. at Sarasota Bay and Shark Bay), associating with related and unrelated females [14], [28], [29], [30], [31]; their associates being mostly dependent upon their reproductive status [5], [14]. For example, in Sarasota Bay (Florida), female strategies appear to be driven by calf protection (from predators and/or conspecifics) with adult females and their calves forming strongly bonded bands [4], [28].

Many studies have shown that bottlenose dolphins have a varied diet and their feeding behaviour may be very flexible [32], [33], [34]. Specializations in the diet [33], [34], [35] and the techniques used to capture prey [11], [12], [13], may also have a role in shaping social structure. However, human activities can have a substantial impact on the social organizations of dolphin groups, for example through changes in the distribution of food resources, which may affect the costs of feeding competition [36]. Anthropogenic food patches in the marine environment, such as aquaculture farms [37] and active trawlers [38], [39] may change the behaviour of bottlenose dolphins through the modification of habitats, changes in predation pressure, and variations of food distribution and availability, which may affect the social interactions and the demography of the dolphin population.

In the Mediterranean, the bottlenose dolphin occurs in coastal habitats [36], [40], [41], [42] and its diet includes several commercial fish species [43]. As fish stocks are generally declining [36], [44] bottlenose dolphins are increasingly in conflict with coastal artisanal fishing [36], [37], [39], including trammel nets, and serious injuries are quite common [45], [46]. At present, the social structure of resident bottlenose dolphin populations in the Mediterranean is poorly understood. The limited information available, however, suggests that aquaculture farms [37], and active trawlers [39], may play an important role in shaping social arrangements.

We began a study on a small population of Mediterranean bottlenose dolphins (Aeolian Archipelago, Southern Italy) in 2005 [47], [48]. In this area the inshore occurrence of the species is related to the complex geomorphology of the volcanic islands [47]. However, the ecology and behaviour of the bottlenose dolphins are likely to be extensively influenced by the food resources found in trammel nets [48]. Dolphin groups are generally smaller in habitats where trawlers operate and foraging groups are smaller than groups of dolphins engaged in other behavioural activities [48].

The purpose of this study is to evaluate the socio-ecological, demographic, and anthropogenic factors that influence the social structure of bottlenose dolphins in the Aeolian Archipelago (2005–2012). Specifically we assessed the role of sex and age composition, individual residency patterns and the temporal stability of association in driving the social organization. We also tested the hypothesis that trammel nets may affect such social patterns, given that dolphin groups were known to interact with this opportunistic food resource [48]. We used measures of association between pairs of individuals (i.e. half-weight association index) [49] to evaluate the social unit composition and cohesion, and statistical methods [50], [51], as well as temporal analysis to describe the temporal stability of dolphins' social structure [52]. As reproductive success is known to have an important role in the sociality of gregarious animals [4], [14], [15], [31] we expect that differences in patterns of associations would occur between males and females. We also expect that dolphins sighted more or less frequently near trammel nets may be socially differentiated, considering that specializations in the diet [32], [34], [35] and techniques used in capturing prey [11], [12], [13] are already known to shape association patterns.

Our results represent the first documentation on the association patterns and social structure for this small bottlenose dolphin population. As the bottlenose dolphin encounter rate and group sizes have been decreasing in recent years, understanding the social structure of this population and how anthropogenic modifications of the environment impact social organizations, may be critical for future conservation efforts.

## Materials and Methods

### Study site and data collection

**Figure 1:**
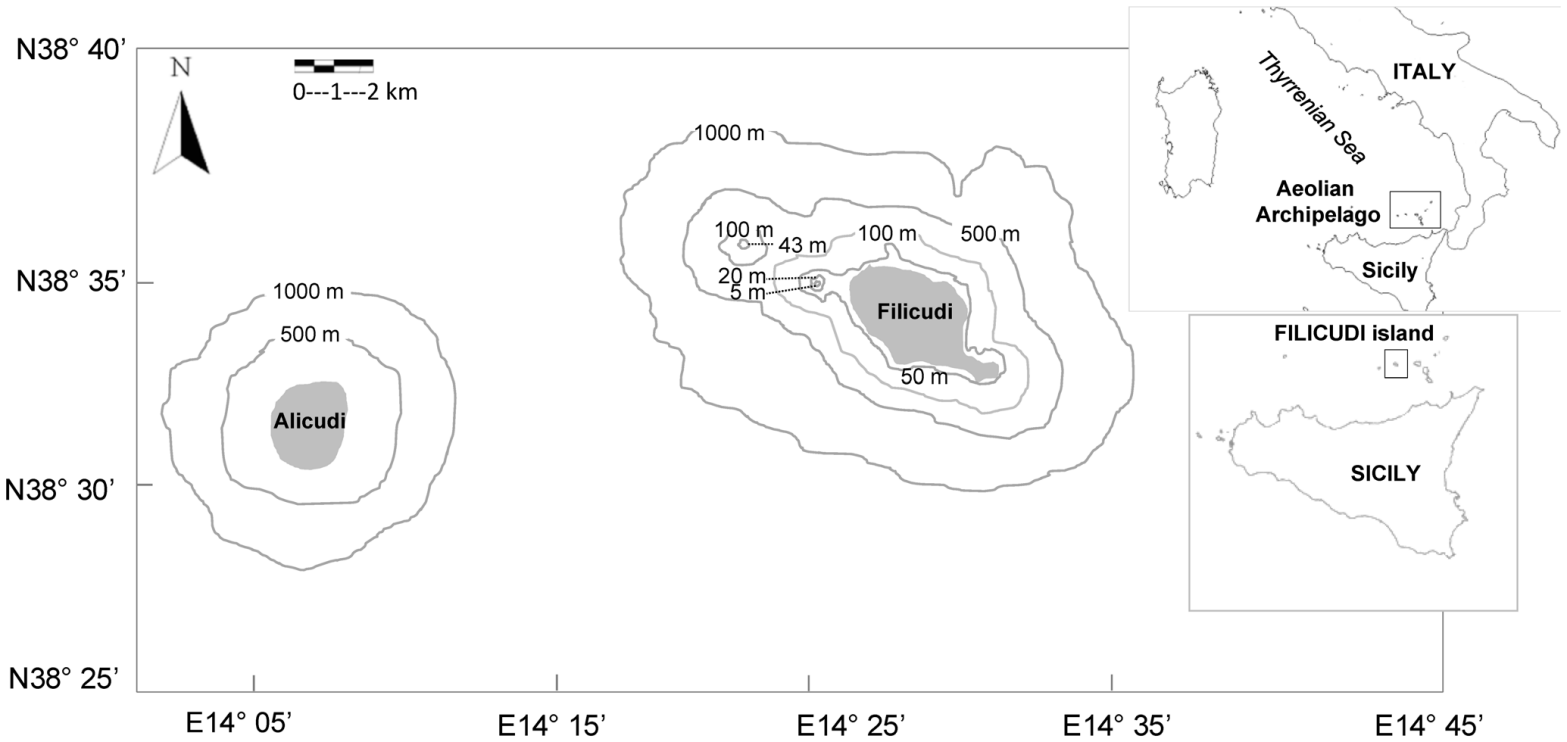
Map of the study area (Filicudi and Alicudi islands, Aeolian Archipelago, Sicily, Italy).

The study area covered 280 km^2^ of coastal waters in Filicudi, one of the seven islands of the Aeolian Archipelago located at 38° 35′ N, 14° 34′ E in the Southern Tyrrhenian Sea (Sicily, Italy) (Fig. 1).

Data on dolphin group sightings covered eight summer periods (2005–2012). Dolphins were sampled from a boat during daily surveys [47]. Surveys were conducted in the morning (from 6.00 a.m. to 14.00 pm), in good light conditions (visibility was more than 300 m) and calm waters (mean  = 1.1; sea state based on Beaufort scale was less than 3), with sea state which did not change between early (mean  = 0.8, SD  = ±0.5, n = 298) and late summer (mean  = 1.7, SD  = ±1.9, n = 159) (Student t, *P* = 0.1) or variations from year to year (Kruskal-Wallis, F = 4.6, *P* = 0.2).

Bottlenose dolphins were sampled using a combination of group follows [53] and photoidentification techniques [54], [55]. The study did not require any permit to observe animals as it was carried out without handling or close contact with animals in the study area. Once sighted, the dolphin groups were slowly approached in order to minimize disturbance and their positions recorded using a hand-held global positioning system (GPS). The first position was recorded only when the boat reached ≤30 m distance from the focal group. For each three-minutes period of sighting, the location of the focal group, its size and the behavioural activity were recorded. We also recorded the position of trammel fishing nets when they were within 100 m of the focal group [48]. Group size was visually determined *in situ*, and later verified by standard photo-id techniques and videos taken during each sighting. Assuming that clustered animals were also interacting [56], we defined associations by membership to the same group, i.e. animals photographed in the same group and moving in the same general direction, interacting or engaged in similar activities were considered to be associated [32]. We included as group members any individuals within 10 m of at least one other dolphin in the group. When more than one group of dolphins was encountered in the same survey, each group was recorded as an independent sighting [57]. When a group split, one of the sub-groups was followed, the choice of group being random and independent of group size and/or activity [57].

Each group member was classified according to its relative size: (1) adults, larger and robust animals, with darker skin color and many marks on the dorsal fin and the body, and often accompanied by a calf [58]; (2) juveniles, less-robust and smaller animals (at least two-thirds the length of adults), usually with less-distinctive nicks, or without nicks in their dorsal fins and not obviously associated with an adult, and (3) calves, smaller animals in close association with an adult and usually without nicks in their dorsal fins. Calves were also identified by behavioural patterns such as surfacing behaviour and swimming in an infant position (in contact underneath the mother) [59].

The sex of the individuals was determined primarily by opportunistic views of the genital region and were later verified by standard photo-id techniques and videos taken during each sighting. Although genetic data are not available to distinguish univocally the sex, the animals showed high site fidelity to the area permitting to collect several photographs for sex determination. Females were sexed when they maintained a close and durable relationship with a calf and were presumed to be mothers. Although bottlenose dolphins show a slight morphometric sexual dimorphism, adult males are known to show a significantly higher number of scars on their dorsal fins than juveniles [60]. Although this feature has not been confirmed for our population, the adult animals with heavy scarring and multiple fin nicks which were never observed in close contact with a calf were classified ‘estimated male’ [30].

### Data organizing

Photographs of dorsal fins were sorted according to standard protocols, using nicks, notches, or scars [55]. We compared good/optimal quality images of distinctive fins with a photo-identification catalog of known individuals. The photographic capture rate was calculated as relative proportion of scarce, good and optimal quality pictures. We calculated the cumulative number of newly-sighted dolphins per survey and the yearly encounter rate (expressed in groups per km) as n/L with n the number of sighted groups and L the effort in km each year [61]. The annual group size was calculated as the mean (± SD) group size each year. We also calculated the mean number (± SD) of dolphins that were not photo-identified each year during the focal follows (not-identified dolphins). We defined residence classes based on the number of sighting of each dolphin. We also calculated the annual residence of each dolphin as the number of individual's sightings each year divided by the total number of sightings each year. Each year, we calculated the percentage of males, females and dolphins of unknown sex and the percentage of calves, juveniles and adults. At the end of the study, all individuals were assigned to an age class according to data acquired from their last sighting. As all dolphins were associated at least once to trammel nets during the study, we calculated the percentage of sightings with and without trammel nets for each dolphin. For each individual, we also calculated the percentage of sightings (1) in early (June–July) and late (August–September) summer, (2) in early (from 6.00 a.m. to 10.00 a.m.) and late (from 10.00 a.m. to 14.00 p.m.) morning and (3) in good (Beaufort scale 0–1) and worse (Beaufort scale 2–3) sea weather conditions. We used the mean group size computed on pooled sightings as cut off value to define small and large groups; for each dolphin we calculated the percentage of sightings within groups with size more and less than this cut off value.

### Quantifying social associations

Coefficients of association were calculated using the half-weight index (HWI), which is defined by the equation 2N/(N_PHDA_+N_PHDB_), where N is the number of times that individual PHDA and PHDB were seen together, and N_PHDA_ and N_PHDB_ are the total number of sightings of each individual [49], [62]. A zero value of HWIs indicates that the pair had never been observed together in the same group while a score of 1 indicates that the animals were always observed in the same group. The HWI accounts for bias from pairs being more likely to be scored when separate than together [62]. We chose the HWI because it allows for comparison with many other studies on bottlenose dolphins [63], [64], [65]. To ensure independence of sampling and avoid serial autocorrelation of sightings, we only used the first sighting when an individual dolphin was sighted more than once in a day [66]. Pooled HWIs (years combined from 2005 to 2012) and one for each combination of sex were calculated for pairs of individuals that were sighted six or more times in the study period. Calves were excluded because of their unique dependent relationship with their mothers [67]. The HWI may be underestimated when not all individuals of a group are identified. For large groups (>8 individuals) only the observations in which at least 80% of the individuals were photo-identified were included in the analysis. We used group membership samples to obtain an association matrix of all individuals [56]. Mean, maximum and minimum HWIs were calculated for each individual.

### Testing for non-random association patterns

Determining whether or not associations are non-random is essential to assess the significance of individual HWIs. We used a permutation test, according to Bejder *et al.* (1998), with modifications following Whitehead *et al.* [68] to test for non-random associations in all data combined against the null hypotheses that dolphins associate randomly [66], [68]. Associations between individuals were tested by randomly permuting groups within sampling periods. In this case the null hypothesis is that there are no preferred or avoided companions (individuals who preferentially group together or avoid one another) given the number of groups each animal is seen in during each sampling period. This method accounts for individuals that are not present in each sampling period due to birth, death and migration, a fact that may occur during the longer time frame of the pooled datasets. If some individuals preferentially associate with other individuals, then the standard deviation (SD) of HWIs should be higher in the real data set than the random data sets. Low means of HWIs, produced by short-term preferences, tended to lower the SD of HWIs and thus mask the presence of long-term preferences. To compensate for this effect, we used the coefficient of variation (CV) of the HWIs as a test for long-term preferences [56], [69]. A significantly higher CV of real HWIs compared to randomized data indicates the presence of long-term preferred companions in the population [56], [69]. The number of random permutations was determined increasing the number of permutations until the p value stabilizes [66]. The resulting *p* values of permutations were not considered as a formal statistical threshold but rather an indication of the strength of evidence for non-random associations; thus, a Bonferroni adjustment was not required. We permuted the associations within daily sampling intervals to remove possible demographic effects [56]. We identified a dyad as having a preferred association when the association index was twice the mean index, including zero values [61]. We chose this arbitrary threshold value because it is approximately twice the expected value when associations were completely random [69]. A Mantel test, using 1,000 permutations, was applied to assess differences in association depending on sex [70]. In addition, a Chi-square test was applied to determine whether the number of dyads on different HWIs classes (selected according to the range and distribution of the HWIs values) significantly differed between sexes.

The calculation of HWI, the permutation test, and the Mantel test were computed using the compiled version of SOCPROG 2.3 [71].

### Network metrics

We calculated three network statistics from the association matrix of individuals using SOCPROG 2.3 [71]. These included (1) the strength, a measure of individuals' gregariousness obtained from the sum of association indices for each individual [72], (2) the clustering coefficient, which represents individual sociality and the proportion of an individual's neighbors that are themselves neighbors [73], and (3) affinity, which represents the weighted mean strength of neighbors and indicates whether individuals strongly connect to individuals that also have strong connections [72]. The variation of the social system was measured using the coefficient of variation of true association indexes (social differentiation), which is an estimate of whether the data reflect a homogeneous society or a socially differentiated population [71].

We used SOCPROG 2.3 [71] to calculate the network statistics for pooled data and obtained mean values and standard errors through the bootstrap method with 1,000 replicates.

### Network analysis

The social organization of the population was graphically represented by a sociogram using SOCPROG 2.3 [71], which does not assume that the society is hierarchically structured.

We also applied a Hierarchical Clustering analysis (average linkage method) to the association coefficients for the entire study period. We obtained a dendrogram where the individuals were arranged on one axis and their degree of association on another [69]. Cluster analysis assumes that the society is structured in a hierarchical fashion with different groups of dolphins clustered together (social units). The relevance of the dendrogram was assessed by means of the correlation coefficient, which is the correlation between the dyads in the association matrix and the level at which the pairs are joined in the dendrogram [69]. A correlation coefficient ≧0.80 indicates a good match and, although it does not indicate a truly hierarchically structured society, it suggests that the model is reasonably consistent with the data [69]. The average-linkage method of clustering was used because it gave the highest correlation coefficient [69] and because very small or large similarities (caused by random error, measurement error or unusual relationships) have less impact on the results than other linkage methods [56]. The association index corresponding to the maximum modularity was used to define community division by clusters [69], [74], [75].

We also explored the factors affecting changes in the association pattern by Principal Component Analysis (PCA) and MANOVA. Specifically we assessed the role of sex and age composition, individual residency patterns and interaction with trammel nets on social organization. The PCA was applied to the HWIs matrix, and the scores on principal components (PC_HWI_) (i.e. the transformed variable values) used as a smaller set of new predictors [47], [76]. The loadings on the PC_HWI_ were used to find the correlation coefficients between HWIs and PC_HWI_. A Student t was applied to test if the PC_HWI_ scores (explaining 80% of variance) were significantly different between sexes and age classes. We also tested the differences between dolphins with mean HWIs larger and smaller than the cut off value for preferred associations. MANOVA was applied to the PC_HWI_ scores with different dependent variables: residence pattern, group size, summer period, sighting time, sea whether conditions and number of sightings with trammel nets. We used the SAS 8.1 software to examine these relationships [77], [78].

### Temporal patterns of association

We calculated the changes in association rates over time within the population using the lagged association rate (LAR; [52]) in relation to time for all relationships (all dyads). The LAR was compared to the null association rate initially to determine whether nonrandom patterns of associations occurred over the entire study period. We developed models of temporal stability [52] using exponential decay and constants to mimic the dynamics of association patterns between pairs of individuals and we fitted the models to the observed data to characterize the social components of the society. The models comprised different combinations of three components, i.e. constant companions (CC, permanent relationships), rapid disassociations (RD) and casual acquaintances (CA; nonpermanent relationships which decay over various time lags), that can last from a few days to several years. For this latter component, the duration of the acquaintances was approximated from the exponent of the exponential function (in days). We tested eight social structure models ranging from societies composed only of constant companionships (in which the association rate in days remains constant through time) to models considering two levels of casual acquaintances and some rapid disassociation, where the constants express the proportion of animals with which an individual associates at rates given by the exponential functions. The model that best described the temporal dynamics of the association patterns was indicated by the smallest quasi-Akaike information criterion (QAIC; [79]). Standard errors for the LAR and parameter estimates were obtained by jackknifing (displayed as 1 standard error interval around the mean).

### Annual residence

We applied a PCA to the annual residence (variable) of each dolphin (statistical unit) to investigate whether association patterns differed significantly among years. Independent principal components (PC_y_) were extracted from the variables and the leading of them, i.e. those which explained more than 80% of variance, loaded by the residence pattern [47], [76]. We used MANOVA to test whether association patterns differed significantly among years; the scores on PC1_y_ and PC2_y_ were applied as independent variables and those on PC1_HWI_ and PC2_HWI_ as dependent variables in the analysis. We used the SAS 8.1 software to examine these relationships [77], [78].

## Results

### Marking, recapturing and residence pattern

A total of 146 sightings were obtained from 2005 to 2012 corresponding to 98.75 hours spent with the dolphins (for sighting: mean  = 36.9 minutes, SD  = 35.2 minutes, range 12–190 minutes). The data used in this paper are based on 100 dolphin sightings that were examined and confirmed by photo-identification. The photographic capture rate was not correlated to the number of newly-sighted dolphins each year (R^2^ = 0.33, *P* = .13). Thirty-four individuals, excluding calves, were well marked and recaptured at least once from 2005 to 2012 (Table 1). The total number of sighted (mean  = 14.25, SD  = ±5.09), newly-sighted (mean  = 4.25, SD  = ±4.37) and re-sighted (mean  = 10.0, SD  = ±5.6) dolphins each year are given in Table 1. We calculated the mean group size computed on pooled sightings (mean  = 5.22, SD  = ±2.21).

**Table 1:** Encounter rate (N/km^2^) (± SD), mean group size (± SD), number of sighted, newly-sighted and re-sighted dolphins and mean not-identified dolphins (± SD) each year.

	2005	2006	2007	2008	2009	2010	2011	2012	Kruskal Wallis test
Mean encounter rate (SD)	0.0256 (0.0052)	0.0256 (0.0061)	0.0265 (0.0053)	0.0052 (0.0042)	0.0065 (0.0033)	0.0045 (0.0043)	0.0036 (0.0018)	0.0006 (0.0013)	*P*<0.0001
Mean group size (SD)	6.20 (2.19)	4.85 (1.37)	5.44 (2.21)	4.58 (3.06)	6.50 (3.72)	6.27 (3.46)	3.93 (1.65)	4.00 (0.81)	*P* = 0.004
Sighted dolphins	10	12	24	14	16	17	14	7	-
Newly- sighted dolphins	10	7	11	2	1	1	1	1	-
Re-sighted dolphins	0	5	13	12	15	16	13	6	-
Mean not-identified (SD)	1.0 (1.7)	2.3 (2.1)	0.5 (0.8)	0.8 (0.7)	0.4 (0.8)	0.2 (1.4)	0	0	*P* = 0.02
Adults (%) (n = 20)	70	100	75	79	75	76	71	43	-
Juveniles (%) (n = 11)	20	0	21	14	19	24	14	57	-
Calves (%) (n = 3)	10	0	4	7	6	0	14	0	-
Females (%) (n = 9)	50	42	33	50	33	45	37	17	-
Males (%) (n = 12)	0	42	38	29	50	35	42	67	-
Unknown sex (%) (n = 13)	50	16	29	21	17	20	21	16	-

In the first column, the percentage of adults, juveniles and calves and the percentage of females, males and dolphins with unknown sex sighted each year. In the last column the results of the test of significance among years for the mean encounter rate, group size and number of not-identified dolphins.

Both the encounter rate and group size decreased from 2005 to 2012 (Table 1). At the end of the study, 59% of dolphins were classified as adults, 32% as juveniles and only 9% as calves (Table 1). Group size was higher with (mean  = 7.91, SD  = ±3.75, range 3–18 individuals) than without (mean  = 3.8, SD  = ±3.1, range 1–12) calves (Student t, *P*<0.0001) in the focal groups. Sex was determined or “estimated” for 61.8% of the photo-identified dolphins: 53% of adult and 9% of juvenile dolphins. Of these, 43% were female and 57% male. Seven females were strongly associated to calves during 25 sightings and the number of calves per group ranged from 1 to 3 with a mean number of calves of 1.2±0.8 when calves were present. The mean number of sightings for individual was 12.2 (SD  = ±9.2, range 1–37). Four residence classes were established according to the number of sightings for each individual: very frequent (>21 times; n = 7), frequent (14–20 times; n = 7), low frequent (10–13 times; n = 5), rare (4–9 times; n = 8) and occasional (1–3 times, n = 7) dolphins. Excluding one male (PHD1), sighted 37 times (the highest value), males (mean  = 14.1, SD  = ±3.9) were sighted less frequently than females (mean  = 21.3, SD  = ±7.9) (Student t, *P*<0.0001). All dolphins, excluding calves, were sighted more frequently in early than in late summer (Student t, *P*<0.0001) with no differences between sexes (Chi-square, *P* = 0.2). Finally, males were sighted more frequently with trammel nets (mean  = 45.4, SD  = ±11.3) than females (mean  = 28.8, SD  = ±8.0) (Student t, *P*<0.0001).

### Social associations

Twenty-three dolphins, 8 females, 11 males and 4 of unknown sex, were used to analyse the association patterns. Two “estimated” males (PHD2 and PHD8) were classified as dolphins of unknown sex in the association analyses, since their high preference for female and calf groups. Pooled HWIs (all years 2005–2012) were calculated for pairs (n = 529, mean  = 0.28, SD  = ±0.16) and one for each combination of sex: male-male (n = 122, mean  = 0.29, SD  = ±0.13, range 0–0.51), female-female (n = 64, mean  = 0.44, SD  = ±0.18, range 0–0.71) and female-male (n = 88, mean  = 0.21, SD  = ±0.12, range 0–0.63).

The observed association matrix of pooled HWIs was randomized 10,000 times with 1,000 flips per permutation with a two-sided significant level of 0.03. Significantly higher SD levels of association (SD  = 0.16268, random SD  = 0.11041, *p*>0.9999) and CV of association indices (CV = 0.70667, random CV = 0.47131, *p*>0.9999) indicates that long-term preferred and avoided companions are present in the population. Fifty-eight preferred associations (11.5%) were identified, i.e. they had association indices which were twice the mean index calculated including zero values. Out of the possible 22, the average individual was observed associated with 21.26 (SD  = ±1.91) individuals. All 23 individuals formed at least one preferred association (i.e. HWI greater than twice the mean) and for each individual, a mean number of 4.57 (SD  = ±2.84, range 1–10) associations was preferred. Seven dolphins had >26% of their associations preferred (6–10 individuals preferred), while only 3 individuals formed one preferred association. The association levels were significantly higher for females than males (Mantel test with 1,000 permutations: *P*<0.001). The number of dyads on 6 classes (selected according to association coefficients values) were significantly higher for females than males (Chi-square test, n = 6, χ^2^ = 5.14, *P*<0.0001). The maximum HWI was significantly different between females (mean  = 0.59±0.13) and males (mean  = 0.49, SD  = ±0.09) (Student t, *P* = 0.02). Associations between pairs were categorized as low (<0.3), moderate (0.3–0.5) and high (>0.5) for pooled data and one for each combination of sex. Of all female-female dyads, 25% (n = 12) had high, 32% (n = 9) moderate and 25% (n = 7) low HWIs. On the contrary, 75% (n = 41) of male-male dyads had low, 22% (n = 12) moderate and only 2 dyads had high HWIs. Only four female-male dyads (4%) had high HWIs, while 19% (n = 17) had moderate and 76% (n = 67) low HWIs. Of all female-NI dyads, 25% (n = 8) had high, 50% (n = 16) moderate and 25% (n = 8) low HWIs. Finally, 7% (n = 3) of male-UN dyads (n = 44) had high, 11% (n = 5) moderate and 81% (n = 36) low HWIs. Two females (PHD3-PHD5) and 3 males (PHD7, PHD11 and PHD17) were sighted at least once with any of the other dolphins (min. HWI >0). The highest HWIs (0.71) were observed for 2 female-female dyads: PHD3-PHD6 and PHD6-PHD16. Fifty avoided associations (9.9%) were identified, i.e. they had association indices with zero value. The number of avoided individuals was smaller for females (mean  = 1.8±1.4; range 1–4) than males (mean  = 2.4±2.6; range 1–9) (Student t, *P* = 0.0007) and 1 juvenile male (PHD13) had the highest number of avoided associations (39.1%).

### Network analysis

Social differentiation was estimated as 0.814 (SD  = ±0.092), indicating a socially well differentiated population (>0.5) [69]. Significant differences in individuals' clustering coefficient, strength and affinity from a random network were assessed using 1,000 permutations. An individual's direct associates were less likely to be connected than expected in a random network (measured by the clustering coefficient: mean  = 0.39, SD  = ±0.05; random mean  = 0.48, random SD  = ±0.02; *P*<0.0001). The individuals had a significantly lower strength (mean  = 5.29, SD  = ±1.56; random mean  = 5.40, random SD  = ±1.25; *P*<0.0001) and higher affinity (mean  = 5.70, SD  = ±0.40; random mean  = 5.68, random SD  = ±0.11; *P*<0.0001) than expected by chance. We found that the dolphins with high degree of strength also have high affinity (*r* = 0.63, *P*<0.001), therefore the individuals preferentially associated with others with similar numbers of associates [69].

**Figure 2:**
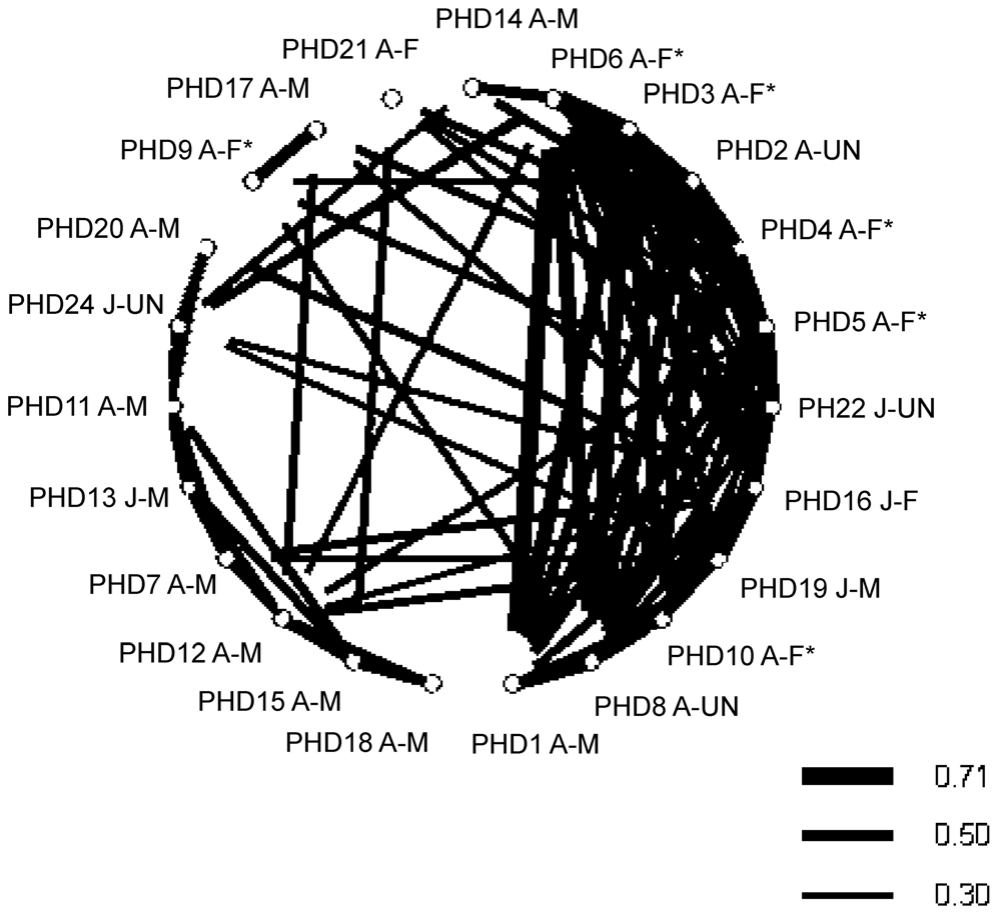
Sociogram showing the associations with HWIs ≧0.30. Lines of increasing thickness correspond to increasing pairwise associations. Study animals are identified by their ID codes (PHDn, n = 1–23), age class (A =  adult, J =  juvenile and C =  calf) and sex (F =  female, M =  male and UN =  unknown sex). Females with calves are marked (F*).

Sociogram (Fig. 2) illustrate the relationships between individuals in all groups (individual dolphins are identified by their ID codes, known sex and age class).

**Figure 3:**
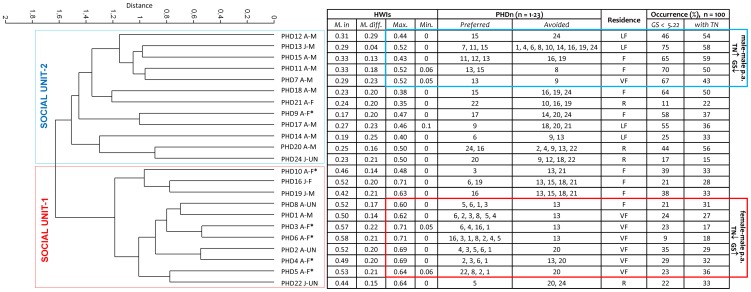
Grouping patterns of 23 individuals. The Clustering eigenvector method identified 2 principal clusters: social units-1 and -2. Dolphin are identified by their ID code (PHDn, n = 1–23), age class (A =  adult, J =  juvenile and C =  calf), and sex (F =  female, F* =  females with calves, M =  male, UN =  unknown sex) Each dolphin is shown with (1) mean HWIs calculated within (M.in) and (2) between (M.diff.) social units, (3) maximum HWI and (4) minimum HWI, (5) preferred and (6) avoided dolphins (min. HWIs), (7) residence pattern (VF: Very frequent, F: Frequent and R: Rare), (8) occurrence (%) in groups with size lower than the mean group size (GS <5.22), (9) occurrence (%) with trammel nets (TN). Male-male and female-male preferred associations (p. a.) are also shown.

The Clustering eigenvector method applied to the association coefficients divided the population into 2 principal clusters (clustering using average linkage; correlation coefficient  = 0.833). Modularity 1 (for gregariousness) was not high enough to represent useful community divisions (modularity  = 0.265) (Fig. 3) [75]. A primary division specifically corresponded to a mixed-sex cluster of 11 animals (social unit-1): 6 females, 2 males and 3 dolphins of unknown sex. Two secondary sub-divisions of this cluster better separated one female (PHD10) and two juveniles from other adult individuals (n = 8). The second cluster corresponded to 12 dolphins (social unit-2) and it was composed of 8 adult males, 2 adult females and 2 juveniles of unknown sex. Four secondary sub-divisions of this cluster better separated 5 males (1 juvenile and 4 adults) from 3 adult males and transitory pairs of male-female dolphins (Fig. 3). Social unit-1 individuals showed higher HWIs (mean  = 0.50, SD  = ±0.12) than those in social unit-2 (mean  = 0.26, SD  = ±0.10) (Student t, *P*<0.0001). In addition, social unit-1 individuals had higher maximum HWIs (mean  = 0.65, SD  = ±0.06) compared to those within social unit-2 (mean  = 0.45, SD  = ±0.06) (Student t, *P*<0.0001). Social unit-2 individuals had a higher number of avoided dolphins (mean  = 3.1, SD  = ±2.2) compared to those in social unit-1 (mean  = 1.8, SD  = ±1.1) (Student t, *P*<0.0001). In general, dolphins in social unit-1 showed higher number of sightings (mean  = 21.4, SD  = ±8.3) than those in social unit-2 (mean  = 13.6, SD  = ±4.7) (Student t, *P*<0.0001). Dolphins in social unit-1 had higher mean number of sightings within groups with size >5.22 (mean  = 74.1, SD  = ±8.7, *P*<0.0001) than those in social unit-2 (mean  = 50.3, SD  = ±21.5; *P* = 0.4). Finally, dolphins in social unit-2 had higher mean number of sightings with trammel nets (mean  = 42.6, SD  = ±14.1, *P* = 0.01) than those in social unit-1 (mean  = 29.2, SD  = ±6.8, *P*<0.0001) (Fig. 3).

**Figure 4:**
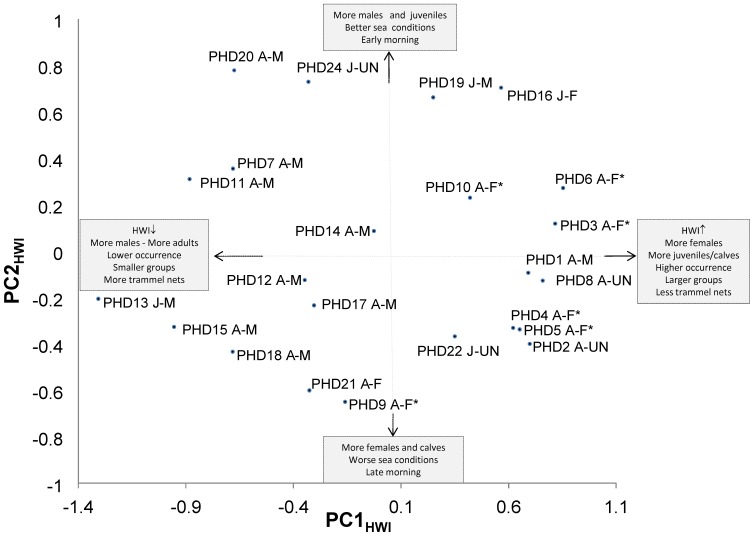
Plot of scores on the principal components, PC1_HWI_ and PC2_HWI_. On the plot, different groups of dolphins are separated according to the explaining variables (age, sex, weather conditions, sighting time, group size and trammel nets). Dolphins with higher (HWI↑) and lower (HWI↓) HWIs than the cut off value for preferred associations are also indicated. Each dolphin is identified by ID code (PHDn, n = 1–23), age class (A =  adult, J =  juvenile and C =  calf) and sex (F =  female, M =  male and UN =  unknown sex). Females with calves are marked (F*).

Eight principal components described 89% of variance with PC1_HWI_ 39% and PC2_HWI_ 17% (Fig. 4). The scores on PC1_HWI_ (Student t, *P*<0.0001) but not on PC2_HWI_ (Student t, *P* = 0.46) were significantly different between dolphins with mean maximum HWI higher and lower than the cut off value for preferred associations (Fig. 4). The annual presence of dolphins was significant on PC1_HWI_ in 2008 (Student t, *P* = 0.007) and on PC2_HWI_ in 2007 (Student t, *P* = 0.008) and 2009 (Student t, *P* = 0.006). The scores on PC1_HWI_ were significantly different between females and males (Student t, *P*<0.0001) and between calves/juveniles and adults (Student t, *P*<0.0001). Both the scores on PC1_HWI_ (Student t, *P*<0.001) and PC2_HWI_ (Student t, *P* = 0.04) were significant in showing differences between females with calves and males with juveniles. On the PC4_HWI_, a different pattern of association which was not dependent on any of the treated variables was identified for the individuals PHD9, PHD14, PHD17, PHD20 and PHD24 (data not shown).

### MANOVA model

The residence pattern was significantly selected by the model (overall MANOVA) (F = 2.64, *P* = 0.006) with the highest values on the PC1_HWI_ (R^2^ = 0.26, *P* = 0.001). No significant difference was observed between summer periods (F = 1.3, *P* = 0.31). A significant difference was observed for group size (F = 18.1, *P*<0.0001) and PC1_HWI_ had the highest values (R^2^ = 0.60, *P*<0.0001), but also PC3_HWI_ (R^2^ = 0.2 , *P* = 0.04). The PC2_HWI_ (R^2^ = 0.25, *P* = 0.014) and PC4_HWI_ (R^2^ = 0.14, *P* = 0.06) were significant for the weather conditions (F = 3.4, *P* = 0.03) while the PC2_HWI_ (R^2^ = 0.29, *P* = 0.006) and PC3_HWI_ (R^2^ = 0.41, *P* = 0.0009) for the sighting time (F = 1.3, *P*<0.0001). Finally, the percentage of interaction with trammel nets (F = 10.9, *P* = 0.0001) was significantly selected on the PC1_HWI_ (R^2^ = 0.56, *P*<0.00001). All these data are graphically represented on Fig. 4.

### Temporal pattern of association

LAR analysis indicated that nonrandom associations within individuals persisted over the entire study period (plot not shown). The LAR appeared to stabilize above the null association rate (i.e., the rate if animals were randomly associating) as time increases, indicating that after group formation, the population maintains stable relationships for multiple days. The lagged association rate of individuals in the population was best explained by a model including three levels of associates (lowest QAIC  = 4311.6252), represented by the equation a_2_+a_3a_ exp ^−(a1*td)^ consisting of CC (long-term, a_2_), CA (short-term, a_3_; individuals associate for a certain length of time and then disassociate) and rapid dissociations (RD; some associates leave very quickly). Associations between dolphins were temporally stable, where individuals had constant companions (a_2_ = 0.38) and casual acquaintances (a_3_ = 0.30) that lasted few days (length of casual acquaintances is a_1_ = 0.33). This suggests that typically individuals remained with a set of associates over periods of days (a mix of CA and CC) and at the end of these days, they rapidly disassociated from all individuals except a moderate number of CC, which share more stable and stronger associations lasting several years. As the smallest difference in QAIC was ▵QAIC  = 6.8, there was no support for any of the other models [79].

### Annual degree of residency

**Figure 5:**
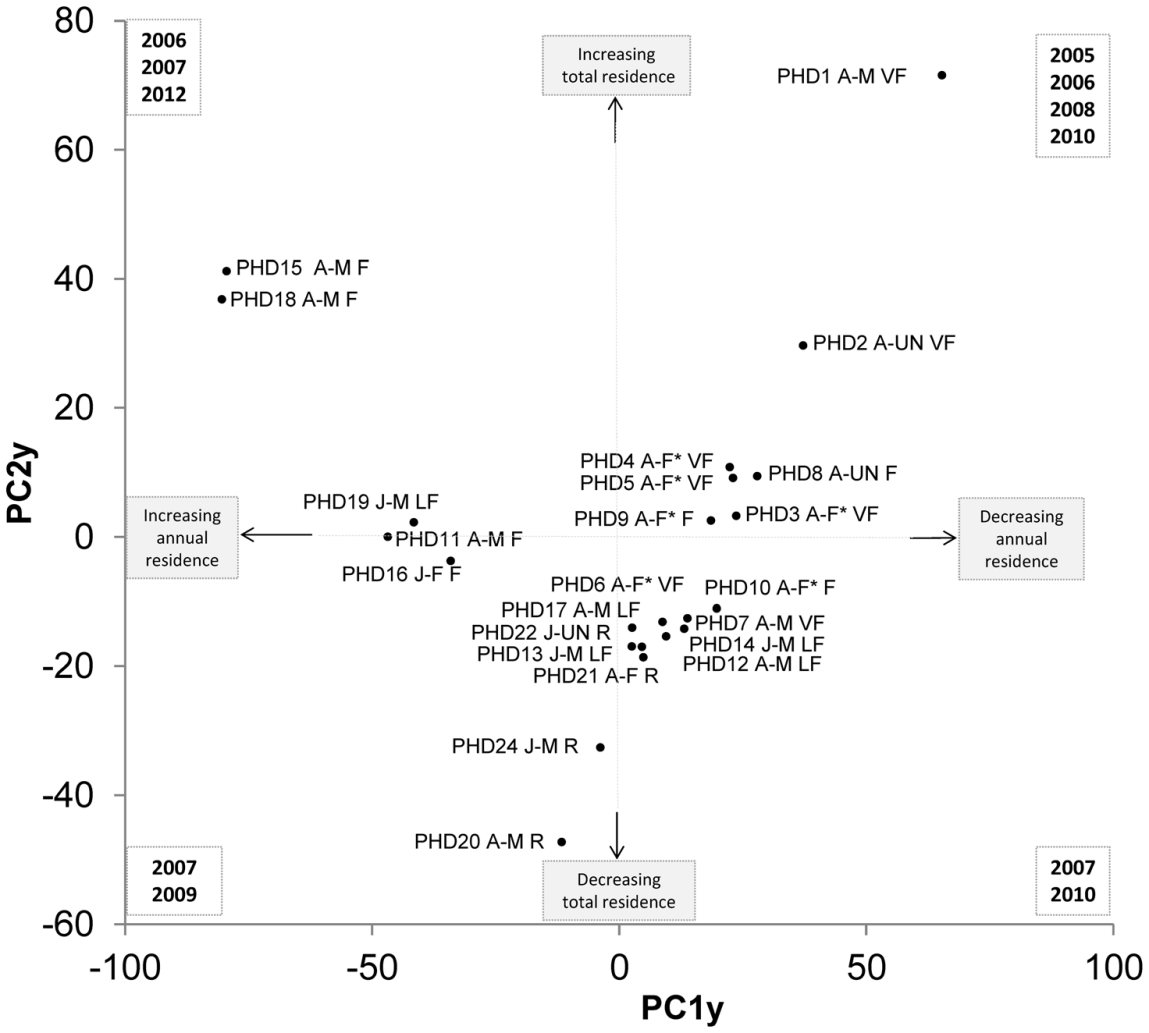
Plot of the principal component scores on PC1y and PC2y representing the annual residence of each dolphin. The dolphins are shown in the plot according to the significant years, i.e. their highest loading values. The residence and association patterns of dolphins are shown in each quarter of the plot according to the test of significance.

Four independent principal components, the PC1y (39.7%), PC2y (21.3%), PC3y (15.2%) and PC4y (9.4%) were calculated, explaining 85.7% of variance. Within a common trend of association pattern, different groups of dolphins were preferentially sighted in particular years (Fig. 5). Using the loading values, we found the annual residence particularly significant in 2005, 2008, 2010 and 2012 on the PC1y while in 2005, 2006, 2009 and 2012 on the PC2y (Fig. 5).

The more frequently sighted dolphins had positive scores on the PC1y and PC2y (Student t, *P*<0.0001). On the contrary, the dolphins with an increasing annual residence pattern had positive scores on the PC1y and negative on the PC2y (Student t, *P*<0.0001). As according to the MANOVA model (F = 2.6, *P* = 0.05), the dolphins with preferred association were represented on the PC1y, with the highest part of information provided by the PC1_HWI_ on the PC1y (R^2^ = 0.25, *P* = 0.01).

## Discussion

### Residence pattern and social structure

Many bottlenose dolphin populations show long-term site fidelity and those in the Aeolian Archipelago appear to be no exception, [28], [65], [80], [81]. Of the 34 dolphins identified in our study, only 2 were sighted once and five <3 times. Seven dolphins were re-sighted repeatedly (21–37 times) in summer months. Three dolphins (PHD1, PHD3, PHD5) were absent for only one year of this study and one (PHD2) for two years. The occurrence of some dolphins (males PHD14 and PHD18, and females PHD10 and PHD21) fluctuated between years and few individuals were not observed for several subsequent years before returning to the study area. At least one dolphin group was sighted each month between 2005 and 2012 suggesting that a core group of animals was permanently resident while several others were passing irregularly to temporarily visit the area from the coastal population. The social organization of the bottlenose dolphin in the Aeolian Archipelago is different to that observed in other populations [4], [5], [24], [26]. We found that the association patterns between dolphins were hierarchically structured, where two mixed-sex social units were subdivided into smaller, temporarily dynamic social groups. The association index, and therefore the partition of clusters, was based on maximum modularity, which was not high enough (modularity  = 0.27) to represent useful community divisions [74], [75], [82]. Furthermore, the cophenetic correlation coefficient value of 0.83 ensured that the hierarchical average linkage cluster analysis of the association data reflects the matrix of interaction rates [82]. Both social units were composed of long-term preferred companions, as observed in previously studied bottlenose dolphin populations [4], [5], [18], [83]; however, the degree of social cohesion, residence pattern and interaction with trammel nets differed considerably between the two social units. The population's society proved to be well differentiated (social differentiation  = 0.814), therefore presenting weak as well as strong relationships between certain individuals. Additionally, we found that the dolphins with high degree of strength also have high affinity. As a result, the important individuals are preferentially linked with each other [69], therefore suggesting the presence of mixing in the population.

### Patterns of association of reproductive females

Females seemed to play an important role in the formation and maintenance of the community social arrangement. In our study, associations between individuals were considerably stronger and temporally more stable in the more resident social unit-1. Six of 8 females (PHD3, PHD4, PHD5, PHD6, PHD10 and PHD16) were clustered within this social unit showing significantly stronger associations (max. HWIs from 0.48–0.71) and high number of preferred companions (from 1–7).

In some bottlenose dolphin populations, females tend to associate more closely with those in a similar reproductive conditions [14], [30], [31]. For example, in Sarasota Bay (Florida) adult females with their calves show stable and high level associations with other females within social clusters named “bands” [4], [65]. Seven females were strongly associated to 8 calves during the study years. Five of these females were clustered in social unit-1 (PHD3, PHD4, PHD5, PHD6 and PHD10), while only one in social unit-2 (PHD9). One calf was strongly associated to a juvenile female (PHD27) sighted only 3 times with social unit-1 individuals, and therefore not used in the analysis. During the study years, 3 calves probably became juveniles (calves of females PHD5, PHD6 and PHD9) while 3 are still calves (calves of females PHD3, PHD4, and PHD27). Two calves probably died (one in 2005 and one in 2009) as they were never re-sighted with their mother (PHD10) in the months after their first sighting. Females in social unit-1 may constitute a small network of mature females with similar reproductive state (i.e. pregnant females or those with similar-aged calves).

The strength of associations between females may be likely to change over years as a result of variable interbirth intervals [31]. Although the same association pattern between females was observed among years, some females were preferentially sighted in particular years. We cannot completely exclude that reproductive females temporarily left the study area to give birth in safer areas. In Shark Bay (Australia), females bottlenose dolphins remained in their natal area and continued to associate with their mothers after conceiving calves [30]. In Sarasota Bay (Florida), females returned to their natal band when their first calf was born, suggesting that bands may be largely composed of maternal relatives [4]. The fluctuations in the annual residence of females with calves, suggest that females may temporarily separate from the other females, returning to the study area after a segregation period.

Potential advantages of group living for female bottlenose dolphins in a similar reproductive status include protection from predators [84], [85] and defense against sexual coercion and infanticide by males [19], [20], [86], [87]. In our study, female bottlenose dolphins had significantly higher preferred associates and lived in larger groups compared to males. As already observed in other bottlenose dolphin populations [4], [30], [81], the group size increased when calves were present and females with calves preferred larger groups than adults only [48]. An interesting result of both the Shark Bay and Sarasota studies is the variation among females' ‘sociability’ with more solitary individuals while others are found in groups [4], [30], [88]. In this study only 2 females (PHD9 and PHD21) showed a more “solitary” behaviour: one female (PHD21) preferred being associated to a mother-juvenile couple (PHD5-PHD22), while the other (PHD9) was strongly associated with 1 adult male (PHD17) but moderately with the other females (mean HWI  = 0.3) and weakly with the other males (mean HWI  = 0.068). The mother-calf bond is the strongest association found in most dolphin communities [4], [30] and kinship may play an important role in determining associations within female bands and band membership [89]. Of 5 juveniles, 3 were clustered within social unit-1 showing higher associations to females (maximum HWI  = 0.71) than males (maximum HWI  = 0.52), while only 2 formed preferred associations with adult males. Preferred associations between female-juvenile pairs were observed in 2 cases (PHD5-PHD22 and PHD6-PHD16) with these juveniles (PHD16 was a female and PHD19 a male) that also showed preferred associations between them (maximum HWI  = 0.63) within social unit-1. Although genetic data are not available to reveal kinship relations, we cannot exclude that some dolphins may arrange associations in relation to kinship and relatives [89], [90].

### Patterns of association of males

The 11 males recorded in this study exhibited striking differences in association pattern and companion preferences suggesting a complex internal structure [4], [15], [18], [91], [92], [93]. We found adult males occurring into 4 general classes: males (PHD1) that formed preferred associations with both males and females; males (PHD7, PHD11, PHD12 and PHD15) that formed preferred associations with 1–3 males but weaker associations with other males and females; males (PHD18) that were solitary or associated for only a short period of time with other males; males (PHD14 and PHD17) that formed preferred male-female pairs only. Our findings differ from those of Shark Bay bottlenose dolphins in which association coefficients of male-male dyads (maximum HWI  = 0.58) match those seen for mother and calf pairs (maximum HWIs  = 0.71) [15], [18]. In general we found male-male associations weaker than most female-female associations (maximum HWIs  = 0.71) but also preferred male-female associations (maximum HWIs  = 0.69). Clustering analysis revealed that males that formed preferred associations with males (from 1–3), generally do not associate or associate weakly with the others. In contrast, “solitary” males were connected for only a short period of time to other males or females. Males that formed preferred associations were consistently observed in the largest groups within social unit-1 and in small groups within social unit-2. On the contrary, males with weaker associations were sighted more frequently in larger groups within social unit-2.

Strong associations may be forced by socio-ecological and demographic factors such as age and sex composition [23], [30], female reproductive status [14] and male-male competition for females [18]. It could be costly for females to frequent mixed-sex groups, except throughout the breeding season [31]. We can suppose that female and male groups associate in the study area during the breeding season and that some male choose to interact with reproductive females forming a distinct but interrelated community. Bottlenose dolphins have a 12-month gestation [88], [94] and, in the Aeolian Archipelago, newborn calves and pregnant females have been observed mainly in spring and early summer, a time that partially coincides with the sightings of the largest mixed-sex groups. As already observed for other bottlenose dolphin populations, we found association of one or more female (single or mother/calf pairs) with one or more males lasting for several days [18], [83], [90], [92]. Most likely, not all the males have the same access to reproductive females. One male (PHD1) clustered in the more resident social unit-1, showed higher access to reproductive females compared to the other males (maximum HWI  = 0.62). Presumably the adult dolphins, PHD2 and PHD8, were males as well; in addition, these dolphins formed preferred associations with at least 1 female (maximum HWIs  = 0.69) but weaker associations with other males, with the exception of PHD1 (maximum HWI  = 0.58).

Some males showed markedly lower residence pattern compared to the other males. This result suggest that these dolphins may be associating in a larger fission–fusion network, which consists of dolphins that appear to temporarily join the network from the coastal population, during the breeding season, and then disappear from the study area [95], [96]. For example, some males (PHD14, PHD17 and PHD20) with a more “solitary” behaviour, formed preferred associations with at least 1 female. These dolphins may not be residents of the area, missing in several years, or have larger home ranges, showing a more irregular residence pattern compared to the other males [95], [96].

### Trammel nets interactions

As bottlenose dolphins are generally believed to have a varied diet [32], [35], [43] specializations in the diet [33], [34], and the techniques used to capture prey [11], [12], [13], might be related to the observed association patterns.

Our results showed that small groups are formed when dolphins forage and feed in the proximity of trammel nets [48]. In addition, the significantly high density of dolphins in the proximity of trammel nets [48] may indicate an adaptation to exploit spatially and temporally variable anthropogenic food resources. We cannot exclude that these dolphins specialized in trammel net foraging, suggesting that this foraging technique may favor a solitary lifestyle, probably because it is easier for dolphins to forage alone and consume small fish while avoiding intraspecific competition [37], [38].

In Sardinia (Italy) the strength of association among preferred associate dolphins was considerably lower when individuals were opportunistically feeding near fish farms [37]. Apparently, the Aeolian population showed a different pattern, where some males (PHD7, PHD11, PHD12, PHD13 and PHD15) interacting with trammel nets formed preferred associations. This structure seems to be similar to that observed in other bottlenose dolphin populations, where individuals showed a more complex social structure composed of distinct communities differently influenced by fishing activities [38], [39], [97], [98]. Intensive trawlers operations and lack of protection of dolphins' habitats in the study area [48] may have changed the distribution of resources required by the dolphins (for food and defense) which may affect the costs of feeding competition and the social organizations of dolphin groups [36], [44], [46]. The *Tursiops truncatus* population in Aeolian Archipelago may be relatively small (only 34 individuals classified based on photoidentification between 2005 and 2012). In addition, the encounter rate and group size are decreasing in the last years and only a core group of animals is resident in the study area; as a result, preferred associations among males may occur as an adaptive response to survive with relatively few associates [7].

We found that 1 juvenile male (PHD13) formed more regularly preferred associations with some males (PHD7, PHD11, PHD15), but avoided many other dolphins. In addition, this male was more frequently sighted with trammel nets and in small groups than the other dolphins. Bottlenose dolphin populations have already been shown to evolve and transmit foraging methods via social learning and local tradition or culture [11], [12], [13]. However, the high bottlenose dolphin feeding requirements [34], may promote the formation of social associations that increases the feeding efficiency via information exchanges. In complex habitats, like the shallow waters of Filicudi island [47], individuals that follow may also benefit from the experience of those that lead them in searching food resources [99]. We cannot exclude that juveniles may attain substantial benefits by cooperating with individuals that have similar foraging preferences [37], [39] or lead them during the foraging activities [99], [100].

The hypotheses that all individuals are gradually shifting to a foraging behaviour associated to trammel nets or that some of them choose not to interact with trammel nets forming a distinct but interrelated social unit cannot be excluded. Behavioural constraints may be essential in driving the development and maintenance of cooperation in this dolphin community [23], [30], [32]. We previously demonstrated a certain degree of spatial segregation between groups of dolphins with different sizes and involved in different activities [48]. We found that female bottlenose dolphins decreased their use of trammel nets habitats and preferred larger groups as they provided more opportunities for resting (defense from predators/disturbance), socializing, and calf care/learning [48]. Large group sizes and high degree of social cohesion for females clustered in social unit-1 could be an indication of greater protection and more efficiency in detecting, deterring or repelling anthropogenic pressures. Given that the local fishermen are known to frighten the dolphins using harpoons or guns in this as in other Mediterranean areas [45], [46], variations in the behavioural patterns between females and males may also create differences in group dynamics between the two social units. Risk of predation has been found to change according to habitat type in other bottlenose dolphin populations [6], [101]. Increasing levels of anthropogenic activities, such as dolphin-watching may also affect group dynamics to some degree [102]. However, as we have no data on the dolphins' social structure prior to the beginning of this study, we cannot unequivocally demonstrate that trammel nets have been the major cause of the existing social structure in the Aeolian Archipelago.

## Conclusions

Our results represent the first documentation on the association patterns and social structure for this endangered bottlenose dolphin population. These findings should be incorporated into population viability analyses in order to contribute to the designing and implementation of appropriate conservation initiatives in the area. Future investigations are required with a larger data set to better understand the primary mechanisms involved during the development and maintenance of these associations. Of particular interest will be to determine the factors, such leadership and dominance, affecting dolphin associations, in addition to the benefits and costs that dolphins incur in cooperating with other dolphins over long periods of time. Our results also improve the current socio-ecological framework around the complex interaction of factors driving *fission–fusion* societies in general.

We confirm that all data underlying the findings in this study are available without restrictions upon request at blasimf@yahoo.com for researchers who meet the criteria for access to confidential data.

## References

[1] Moss CJ, Poole JH (1983) Relationships and social structure of African elephants. In: Hinde RAeditor. Primate social relationships. An integrated approach. Oxford: Blackwell Scientific Publications. pp.315–325.

[2] Wrangham RW, Rubenstein DI (1986) Social evolution in birds and mammals. In: Rubenstein DI, Wrangham RWeditors. Ecological aspects of social evolution. Princeton: Princeton University Press.

[3] White FJ (1992) Pygmy chimpanzee social organization: variation with party size and between study sites. Am J Primatol 26:203–214. 10.1002/ajp.1350260306 31948159

[4] Wells RS, Scott MD, Irvine AB (1987) The social structure of free ranging bottlenose dolphins. In: Genoways HHeditor. Curr Mamm Vol 1. New York: Plenum Press. pp.247–305.

[5] Connor RC, Wells RS, Mann J, Read AJ (2000) The bottlenose dolphin: social relationships in a fission–fusion society. In: Mann J, Connor RC, Tyack PL, Whitehead Heditors. Cetacean societies. Chicago: University of Chicago Press. pp.91–126.

[6] Heithaus MR, Dill LM (2002) Food availability and tiger shark predation risk influences bottlenose dolphin habitat use. Ecology 83:480–491.

[7] Lusseau D, Schneider K, Boisseau OJ, Haase P, Slooten E, et al (2003) The bottlenose dolphin community of Doubtful Sound features a large proportion of long-lasting associations. Can geographic isolation explain this unique trait? Behav Ecol Sociobiol 54:396–405.

[8] Wiszniewski J, Beheregaray LB, Allen SJ, Möller LM (2010) Environmental and social influences on the genetic structure of bottlenose dolphins (*Tursiops aduncus*) of south-eastern Australia. Conservation Genetics 11:1405–1419.

[9] Wiszniewski J, Allen SJ, Möller LM (2009) Social cohesion in a hierarchically structured embayment population of Indo-Pacific bottlenose dolphins. Anim Behav 77:1449–1457.

[10] Gowans S, Würsig B, Karczmarski L (2008) The social structure and strategies of dephinids: predictions based on an ecological framework. Adv Mar Biol 53:195–294. 10.1016/S0065-2881(07)53003-8 17936137

[11] Krützen M, Mann J, Heithaus MR, Connor RC, Bejder L, et al (2005) Cultural transmission of tool use in bottlenose dolphins. Proc Natl Acad Sci U S A 102:8939–8943. 15947077 10.1073/pnas.0500232102 PMC1157020

[12] Sargeant BL, Mann J, Berggren P, Krützen M (2005) Specialization and development of beach hunting, a rare foraging behavior, by wild bottlenose dolphins (*Tursiops sp*.). Can J Zool 83:1400–1410.

[13] Daura-Jorge FG, Cantor M, Ingram SN, Lusseau D, Simones-Lopes PC (2012) The structure of a bottlenose dolphin society is coupled to a unique foraging cooperation with artisanal fishermen. Biol Lett 8:702–705. 22552635 10.1098/rsbl.2012.0174 PMC3440962

[14] Möller LM, Harcourt AH (2008) Shared reproductive state enhances female associations in dolphins. Res Lett Ecol 1:1–5.

[15] Connor RC, Smolker RA, Richards AF (1992a) Dolphin alliances and coalitions. In: Harcourt AH, de Waal FBMeditors. Coalitions and alliances in humans and other animals. Oxford: Oxford University Press. pp.415–443.

[16] Connor RC, Smolker RA, Richards AF (1992b) Two levels of alliance formation among bottlenose dolphins (*Tursiops sp*.). Proc Natl Acad Sci U S A 89:987–990. 11607275 10.1073/pnas.89.3.987 PMC48370

[17] Connor RC, Heithaus RM, Barre LM (1999) Superalliance of bottlenose dolphins. Nature 371:571–572.

[18] Connor RC, Heithaus MR. Barre LM (2001) Complex social structure, alliance stability and mating access in a bottlenose dolphin ‘super-alliance’. Proc R Soc Lond B 268:263–267. 10.1098/rspb.2000.1357 PMC1088601 11217896

[19] Hrdy SB (1979) Infanticide among animals: a review, classification, and examination of the implications for the reproductive strategies of females. Ethol Sociobiol 1:13–40.

[20] Ross HM, Wilson B (1996) Violent interactions between bottlenose dolphins and harbour porpoises. Proc R Soc Lond B 263:283–286. 10.1098/rspb.1998.0414 PMC1689180 9699310

[21] Dunn DG, Barco SG, Pabst DA, McLellan WA (2002) Evidence for infanticide in bottlenose dolphins of the Western North Atlantic. J Wildl Dis 38:505–510. 12238367 10.7589/0090-3558-38.3.505

[22] Krause J, Ruxton GD (2002) Living in Groups. Oxford: Oxford University Press.

[23] Fury CA, Ruckstuhl KE, Harrison PL (2013) Spatial and Social Sexual Segregation Patterns in Indo- Pacific Bottlenose Dolphins (*Tursiops aduncus*). Plos One 8(1).. 10.1371/journal.pone.0052987 PMC3541364 23326370

[24] Wilson B, Thompson P, Hammond P (1993) An examination of the social structure of a resident group of bottle-nosed dolphins (*Tursiops truncatus*) in the Moray Firth, N.E. Scotland. European Research on Cetaceans 7:54–56.

[25] Bearzi G, Notarbartolo di Sciara G, Politi E (1997) Social ecology of bottlenose dolphins in the Kvarnerić (Northern Adriatic sea). Mar Mamm Sci 13(4):650–668.

[26] Eisfeld SM (2003) The social affiliation and group composition of bottlenose dolphins (*Tursiops truncatus*) in the outer southern Moray Firth, NE Scotland. [Master thesis]. Bangor: University of Wales. 85 p.

[27] Foley A, McGrath D, Berrow S, Gerritsen H (2010) Social structure within the bottlenose dolphin (*Tursiops truncatus*) population in the Shannon Estuary, Ireland. Aquatic Mammals 36(4):372–381.

[28] Wells RS (1991) The role of long-term study in understanding the social structure of a bottlenose dolphin community. In: Pryor K, Norris KSeditors. Dolphin societies: discoveries and puzzles. Berkeley: University of California Press. pp.199–225.

[29] Duffield DA, Wells RS (1991) The combined application of chromosome protein and molecular data for the investigation of social unit structure and dynamics in *Tursiops truncatus* . Rep Intl Whal Commiss (special issue) 13:155–170.

[30] Smolker RA, Richards AF, Connor RC, Pepper JW (1992) Sex differences in patterns of association among Indian Ocean bottlenose dolphins. Behaviour 123:38–69.

[31] Mann J, Connor RC, Barre LM, Heithaus MR (2000) Female reproductive success in bottlenose dolphins (*Tursiops sp*.): life history, habitat, provisioning, and group-size effects. Behav Ecol 11(2):210–219.

[32] Shane SH, Randall SW, Würsig B (1986) Ecology, behavior and social organization of the bottlenose dolphin: a review. Mar Mammal Sci 2(1):34–63.

[33] Barros NB, Odell DK (1995) Bottlenose dolphin feeding and interactions with fisheries in the Indian River Lagoon system. Bull Mar Sci 57:278–285.

[34] Barros NB, Wells RS (1998) Prey and feeding patterns of resident bottlenose dolphins (*Tursiops truncatus*) in Sarasota Bay, Florida. J Mamm 79:1045–1059.

[35] Mioković D, Kovacić D, Pribanić S (1999) Stomach contents analysis of one bottlenose dolphin (*Tursiops truncatus*) from the Adriatic Sea. Nat Croat 8:61–65.

[36] Bearzi G, Fortuna CM, Reeves RR (2009) Ecology and conservation of common bottlenose dolphins *Tursiops truncatus* in the Mediterranean Sea. Mammal Review 39:92–123.

[37] Díaz López B, Bernal Shirai JA (2008) Marine aquaculture and bottlenose dolphins' (*Tursiops truncatus*) social structure. Behav Ecol Sociobiol 62:887–894.

[38] Chilvers BL, Corkeron PJ (2001) Trawling and bottlenose dolphins' social structure. Proc R Soc Lond B Bio 268:1901–1905. 10.1098/rspb.2001.1732 PMC1088825 11564345

[39] Pace DS, Pulcini M, Triossi F (2011) Anthropogenic food patches and association patterns of *Tursiops truncatus* at Lampedusa island, Italy. Behav Ecol 23(2):254–264.

[40] Bearzi G, Politi E, Agazzi S, Bruno S, Costa M, et al (2005) Occurrence and present status of coastal dolphins (*Delphinus delphis* and *Tursiops truncatus*) in the eastern Ionian Sea. Aquatic Conservation: Marine and Freshwater Ecosystems 15:243–257.

[41] Forcada J, Gazo M, Aguilar A</initiql>, Gonzalvo J, Fernandez-Contreras M (2004) Bottlenose dolphin abundance in the NW Muditerranean: addressing heterogeneity in distribution. Mar Ecol Prog Ser 275:275–287.

[42] De Smgura AG, Hammond PS, Raga JA (200) Influence of environmental factors on small cetacean distribution mn the Spanish Mediterranean. J Mar Biol Assoc UK 88:1185–1192.

[43] Blanco O, Salomon O, Raga JA (2001) Diet of bottlenose dolphin (*Tursiops truncqtus*) in the western Mediterranean Sea. J Mar Biol Ass UK 81:1053–1058.

[44] Bearzi G, Politi E, Agazzi S, Azzullino A (2006) Prey depletion caused by overfishing and the decline of marine megafauna in eastern Ionian Sea coastal waters (central Mediterranean). Biol Cons 127:373–382.

[45] Lauriano G, Fortuna CM, Moltedo G, Notarbartolo di Sciara G (2004) Interaction between common bottlenose dolphin (*Tursiops truncatus*) and the artisanal fishery in Asinara island National Park (Sardinia): assessment of catch damage and economic loss. J Cetacean Res Manage 6:165–173.

[46] Lauriano G, Caramanna L, Scarno M, Andaloro F (2009) An overview of dolphin depredation in Italian artisanal fisheries. J Mar Biol Assoc UK Special Issue 05(89):921–929.

[47] Blasi MF, Boitani L (2012) Modelling fine-scale distribution of the bottlenose dolphin *Tursiops truncatus* using physiographic features on Filicudi (Southern Thyrrenian Sea, Italy). End Spec Res 17:269–288.

[48] Blasi MF, Boitani L (2013) Opportunistic feeding in trammel nets can affect bottlenose dolphin (*Tursiops truncatus*) group size in Aeolian Archipelago (Southern Italy). European Research on Cetacean E06:255.

[49] Hinde RA (1976) Interactions, relationships and social structure. Man 11:1–17.

[50] Whitehead H (1997) Analysing animal social structure Anim Behav. 53:1053–1067.

[51] Whitehead H (1999) Testing association patterns of social animals. Anim Behav 57:26–29. 10.1006/anbe.1999.1099 10373270

[52] Whitehead H (1995) Investigating structure and temporal scale in social organizations using identified individuals. Behav Ecol 6:199–208.

[53] Altmann J (1974) Observational study of behaviour: Sampling methods. Behaviour 49:227–265. 4597405 10.1163/156853974x00534

[54] Würsig B, Würsig M (1977) The photographic determination of group size, composition and stability of coastal purpoises (*Tursiops truncatus*). Science 198:755–756.

[55] Würsig B, Jefferson TA (1990) Methods of photo-identification for small cetaceans. In: Hammond PS, Mizroch SA, Donovan GP, editors. Individual recognition of cetaceans: use of photo-identification and other techniques to estimate population parameters. Report of the International Whaling Commission Special Issue 12:43–52.

[56] Whitehead H, Dufault S (1999) Techniques for analyzing vertebrate social structure using identified individuals: review and recommendations. Adv Stud Behav 28:33–74.

[57] Mann J (1999) Behavioural sampling methods for cetaceans: a review and critique. Mar Mammal Sci 15:102–122.

[58] Hersh SL, Duffield DA (1990) Distinction between northwest Atlantic offshore and coastal bottlenose dolphins based on hemoglobin profile and morphometry. In: Leatherwood S, Reeves RReditors. The bottlenose dolphin. San Diego: Academic Press. pp.129–139.

[59] Grellier K, Hammond PS, Wilson B, Sanders-Reed CA, Thompson PM (2003) Use of photo-identification data to quantify mother-calf association patterns in bottlenose dolphins. Can J Zool 81:1421–1427.

[60] Tolley KA, Read AJ, Wells RS, Urian KW, Scott MD, et al (1995) Sexual dimorphism in wild bottlenose dolphins (*Tursiops truncatus*) from Sarasota, Florida. J Mamm 76:1190–1198.

[61] Gero S, Bejder L, Whitehead H, Mann J, Connor RC (2005) Behaviourally specific preferred associations in bottlenose dolphins, *Tursiops spp* . Can J Zool 83:1566–1573.

[62] Cairns SJ, Schwager SJ (1987) A comparison of association indices. Anim Behav 35:1454–1469.

[63] Bräger S, Würsig B, Acevedo A, Henningsen T (1994) Association patterns of bottlenose dolphins (*Tursiops truncatus*) in Galveston Bay, Texas. J Mammal 75(2):431–437.

[64] Ansmann IC, Parra GJ, Chilvers BL, Lanyon JM (2013) Dolphins restructure social system after reduction of commercial fisheries. Anim Behav 84:575–581.

[65] Wells RS (2003) Dolphin social complexity: lessons from long-term study and life history. In: de Waal FBM, Tyack PLeditors. Animal Social Complexity: Intelligence, Culture, and Individualized Societies. Cambridge & London: Harvard University Press. pp.32–56.

[66] Bëjder L, Fletcher D, Bräger S (1998) A method for testing association patterns of social animals. Anim Behav 56:719–725. 9784222 10.1006/anbe.1998.0802

[67] Mann J, Smuts B (1998) Natal attraction: allomaternal care and mother-infant separations in wild bottlenose dolphins. Anim Behav 55:1097–1113. 9632497 10.1006/anbe.1997.0637

[68] Whitehead H, Bejder L, Ottensmeyer AC (2005) Testing association patterns: issues arising and extensions. Anim Behav 69:1–6.

[69] Whitehead H (2008) Analyzing animal societies: quantitative methods for vertebrate social analysis. Chicago (IL): University of Chicago Press.

[70] Mantel N (1967) The detection of disease clustering and a generalized regression approach. Cancer Res 27:209–220. 6018555

[71] Whitehead H (2008) Compiled version of SOCPROG 2.3 (computer program) and manual “Programs for analyzing social structure”. http://myweb.dal.ca/hwhitehe/social.htm. pp.73.

[72] Barthelemy M, Barrat A, Pastor-Satorras R, Vespignani A (2005) Characterization and modeling of weighted networks. Physica A 346:34–43.

[73] Holme P, Park SM, Kim BJ, Edling CR (2007) Korean university life in a network perspective: dynamics of a large affiliation network. Physica A 373:821–830.

[74] Newman MEJ (2004) Analysis of weighted networks. Phys Rev E 70:056131. 10.1103/PhysRevE.70.056131 15600716

[75] Newman MEJ (2006) Modularity and community structure in networks. Proc Natl Acad Sci U S A 103(23):8577–8582. 16723398 10.1073/pnas.0601602103 PMC1482622

[76] Jolliffe IT (1972) Discarding variables in a principal component analysis. II. Real data. Appl Stat 21:160–173.

[77] Sall JP (1981) SAS Regression Applications, Revised Edition, SAS Technical Report A-102. Cary, NC: SAS Institute Inc.

[78] Freund RJ, Littell RC (1986) SAS System for Regression, 1986 Edition, Cary, NC: SAS Institute Inc.

[79] Whitehead H (2007) Selection of models of lagged identification rates and lagged association rates using AIC and QAIC. Commun Stat Simul Comput 36:1233–1246.

[80] Scott MD, Wells RS, Irvine AB (1990) A long-term study of bottlenose dolphins on the west coast of Florida. In: Leatherwood S, Reeves RReditors. The Bottlenose Dolphin. San Diego: Academic Press. pp.235–244.

[81] Möller LM, Allen SJ, Harcourt RG (2002) Group characteristics, site fidelity and seasonal abundance of bottlenose dolphins *Tursiops aduncus* in Jervis Bay and Port Stephens, south-eastern Australia. Aust Mamm 24:11–21.

[82] Whitehead H (2009) SOCPROG programs: Analyzing animal social structures. Behav Ecol and Soc 63:765–778 http://dx.doi.org/10.1007/s00265-008-0697-y

[83] Owen EC, Wells RS, Hofmann S (2002) Ranging and association patterns of paired and unpaired adult male Atlantic bottlenose dolphins, *Tursiops truncatus*, in Sarasota, Florida, provide no evidence for alternative male strategies. Can J Zool 80:2072–2089.

[84] Corkeron PJ, Morris RJ, Bryden MM (1987) Interactions between bottlenose dolphins and sharks in Moreton Bay, Queensland. Aquatic Mammals 13:109–113.

[85] Cockcroft VG, Cliff G, Ross GJB (1989) Shark predation on Indian Ocean bottlenose dolphins (*Tursiops truncatus*) off Natal, South Africa. S Afr J Zool 24:305–310.

[86] Patterson AP, Reid RJ, Wilson BK, Grellier HM, Rossand PM (1998) Thompson Evidence for infanticide in bottlenose dolphins: an explanation for violent interactions with harbour porpoises? Proc R Soc Lond B 265:167–1170. 10.1098/rspb.1998.0414 PMC1689180 9699310

[87] Fearnbach H, Durban J, Parsons K, Claridge D (2012) Seasonality of calving and predation risk in bottlenose dolphins on Little Bahama Bank. Mar Mamm Sci 28(2):402–411.

[88] Connor RC, Richards AF, Smolker RA, Mann J (1996) Patterns of female attractiveness in Indian Ocean bottlenose dolphins. Behaviour 133:37–69.

[89] Möller LM, Beheregaray LB, Allen SJ, Harcourt RG (2006) Association patterns and kinship in female Indo-Pacific bottlenose dolphins (*Tursiops aduncus*) of southeastern Australia. Behav Ecol Soc 61(1):109–117.

[90] Möller LM, Beheregaray LB, Harcourt RG, Krützen M (2001) Alliance membership and kinship in wild male bottlenose dolphins (*Tursiops aduncus*) of southeastern Australia. Proc R Soc Lond B Bio 268:1941–1947. 10.1098/rspb.2001.1756 PMC1088832 11564352

[91] Wiszniewski J, Brown C, Möller LM (2012) Complex patterns of male alliance formation in a dolphin social network. J Mamm 93(1):239–250.

[92] Parsons KM, Durban JW, Claridge DE, Balcomb KC, Noble LR, et al (2003) Kinship as a basis for alliance formation between male bottlenose dolphins, *Tursiops trunactus*, in the Bahamas. Anim Behav 66:185–194.

[93] Rogers CA, Brunnick BJ, Herzing DL, Baldwin JD (2004) The social structure of bottlenose dolphins, *Tursiops truncatus*, in the Bahamas. Mar Mamm Sci 20:688–708.

[94] Schroeder JP (1990) Breeding bottlenose dolphins in captivity. In Leatherwood S, Reeves RR, editors. The bottlenose dolphin. New York: Academic Press. pp.435–446.

[95] Möller LM, Beheregaray LB (2004) Genetic evidence for sex-biased dispersal in resident bottlenose dolphins (*Tursiops aduncus*). Mol Ecol 13:1607–1612. 15140103 10.1111/j.1365-294X.2004.02137.x

[96] Natoli A, Birkun A, Aguilar A, Lopez A, Hoelzel AR (2005) Habitat structure and the dispersal of male and female bottlenose dolphins (*Tursiops truncatus*). Proc R Soc Lond B 272:1217–1226. 10.1098/rspb.2005.3076 PMC1564106 16024385

[97] Corkeron PJ, Bryden MM, Hedstrom KE (1990) Feeding by bottlenose dolphins in association with trawling operations in Moreton Bay, Australia. In: Leatherwood S, Reeves RReditors. The bottlenose dolphin. San Diego (CA): Academic Press, Inc. pp.329–336.

[98] Chilvers BL, Corkeron PJ (2003) Influence of trawling on the behavior and spatial distribution of Indo-Pacific bottlenose dolphins (*Tursiops aduncus*) in Moreton Bay, Australia. Can J Zool 81:1947–1955.

[99] Lewis JS, Wartzok D, Heithaus MR (2011) Highly dynamic fission-fusion species can exhibit leadership when traveling. Behav Ecol Sociobiol 65:1061–1069.

[100] Lewis JS, Wartzok D, Heithaus M, Krützen M (2013) Could Relatedness Help Explain Why Individuals Lead in Bottlenose Dolphin Groups? PLoS ONE 8(3). 10.1371/journal.pone.0058162 PMC3596398 23516445

[101] Croft D, Morrell L, Wade A, Piyapong C, Ioannou C (2006) Predation risk as a driving force for sexual segregation: a cross-population comparison. Amer Nat 167:867–878. 16649156 10.1086/504853

[102] Allen SJ (2005) Management of bottlenose dolphins (*Tursiops aduncus*) exposed to tourism in Port Stephens, N.S.W, Australia. Master thesis, Macquarie University, Sydney.

